# The Mortality Risk and Socioeconomic Vulnerability Associated with High and Low Temperature in Hong Kong

**DOI:** 10.3390/ijerph17197326

**Published:** 2020-10-07

**Authors:** Sida Liu, Emily Yang Ying Chan, William Bernard Goggins, Zhe Huang

**Affiliations:** 1 Collaborating Centre for Oxford University and CUHK for Disaster and Medical Humanitarian Response (CCOUC), The Chinese University of Hong Kong, Hong Kong SAR, China; liusida2008@gmail.com (S.L.); huangzhe@cuhk.edu.hk (Z.H.); 2 JC School of Public Health and Primary Care, Faculty of Medicine, The Chinese University of Hong Kong, Hong Kong SAR, China; wgoggins@cuhk.edu.hk

**Keywords:** H-EDRM, climate change, extreme temperature, climate change, socioeconomic vulnerability, health disparities

## Abstract

(1) Background: The adverse health effect associated with extreme temperature has been extensively reported in the current literature. Some also found that temperature effect may vary among the population with different socioeconomic status (SES), but found inconsistent results. Previous studies on the socioeconomic vulnerability of temperature effect were mainly achieved by multi-city or country analysis, but the large heterogeneity between cities may introduce additional bias to the estimation. The linkage between death registry and census in Hong Kong allows us to perform a city-wide analysis in which the study population shares virtually the same cultural, lifestyle and policy environment. This study aims to examine and compare the high and low temperature on morality in Hong Kong, a city with a subtropical climate and address a key research question of whether the extreme high and low temperature disproportionally affects population with lower SES. (2) Methods: Poisson-generalized additive models and distributed-lagged nonlinear models were used to examine the association between daily mortality and daily mean temperature between 2007–2015 with other meteorological and confounding factors controlled. Death registry was linked with small area census and area-level median household income was used as the proxy for socioeconomic status. (3) Results: 362,957 deaths during the study period were included in the analysis. The minimum mortality temperature was found to be 28.9 °C (82nd percentile). With a subtropical climate, the low temperature has a stronger effect than the high temperature on non-accidental, cardiovascular, respiratory and cancer deaths in Hong Kong. The hot effect was more pronounced in the first few days, while cold effect tended to last up to three weeks. Significant heat effect was only observed in the lower SES groups, whilst the extreme low temperature was associated with significantly higher mortality risk across all SES groups. The older population were susceptible to extreme temperature, especially for cold. (4) Conclusions: This study raised the concern of cold-related health impact in the subtropical region. Compared with high temperature, low temperature may be considered a universal hazard to the entire population in Hong Kong rather than only disproportionally affecting people with lower SES. Future public health policy should reconsider the strategy at both individual and community levels to reduce temperature-related mortality.

## 1. Introduction

The adverse effect of extreme temperature on human health has been intensively reported during the last decade [1,2]. Epidemiological evidence shows that both high and low temperature were associated with increased mortality and morbidity in western, Asian and African countries [3,4,5]. With the effect of climate change, extreme weather events are expected to be more frequent and intensive in the future, which may impose a significant health burden in the years to come [6]. Previous studies have also identified that temperature may pose different health impact to population with different characteristics, some risk factors related to the vulnerability of extreme temperature include pre-existing diseases, housing, behavior as well as socioeconomic status (SES) [7,8,9]. 

The association between health inequities and socioeconomic disparities have been well established [10,11,12]. A number of research revealed that such disparities were also existed for health effect induced by temperature [13,14]. Current research has shown inconsistent results on whether socioeconomic disadvantages were associated with higher temperature-related health risk, especially for cold weather [7,8,14,15]. Previous studies of such kind were mainly conducted by city or country-level comparison [8,16,17]. This may be problematic since other confounding factors such as lifestyle, behavior, temperature-related policy and acclimation are difficult to properly controlled. An intra-city analysis has advantages to minimize the effect of other confounding factors. This was rarely conducted in previously due to methodological challenges that SES data are often absent in retrospective health-related data in most countries.

In Hong Kong, the mortality registry can be lined with area-level census data, which provide an opportunity to perform such city-wide analysis in which the study population shares virtually the same cultural, lifestyle and policy environment. Hong Kong is a developed metropolis with one of the largest income disparities in the world. The Gini coefficient was reported to be 0.539 for original and 0.473 for after social benefit transfer and it continues growing in the last two decades [18]. The large income disparity gap provides a good example with which to investigate the modifying effect of SES on temperature. Many mortality and morbidity related to extreme temperature are predictable and preventable [19]. As highlighted in UNISDR Sendai Framework and WHO health emergency disaster risk management (Health-EDRM), understanding the health risk and vulnerable population are priorities to reduce the exposure and adverse health consequences that are associated with disaster and environmental hazards [20]. In this study, we aimed to (1) examine and compare morality risk associated with high and low temperature in the subtropical city and (2) to address a key research question of whether the extreme high and low temperature disproportionally affects population with lower SES.

## 2. Materials and Methods

### 2.1. Environmental Data

Meteorological data including mean daily temperature, relative humidity and atmospheric pressure from 1 January 2007 to 31 December 2015 were obtained from Hong Kong Observatory, which is located at the center of the urban area. To adjust for the potential confounding effect of air pollution, daily mean air pollutant data including nitrogen dioxide (NO_2_), Sulphur dioxide (SO_2_), Ozone (O_3_) and particulate matter less than 2.5 μm aerodynamic diameter (PM_2.5_) of the same period were collected from the Hong Kong Environmental Protection Department. The daily air pollution variables were calculated by taking an average of ten general stations.

### 2.2. Mortality Data

The corresponding daily non-accidental mortality data were collected from the Hong Kong Census and Statistics Department for analysis. The data also contain the cause of death, gender and age for stratified analysis. Cause of death was also analyzed which classified according to International Classification of Diseases, Tenth Revision (ICD-10) non-accidental (A00-T99, Z00-Z99), cardiovascular (I00-I99) and respiratory (J00-J98) diseases and cancer (C00-D48).

### 2.3. Socioeconomic Status Data

**Figure 1:**
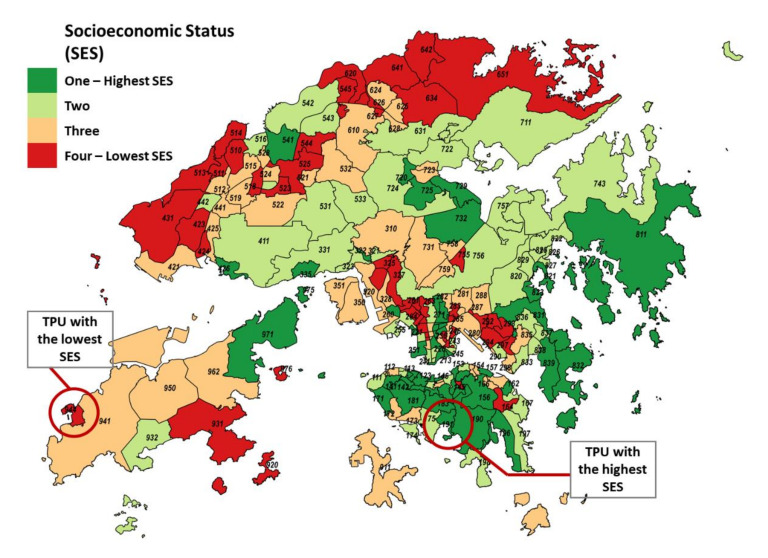
The geographical distribution of socioeconomic status (SES) groups by median monthly household income of tertiary planning units (TPUs) in Hong Kong, where One = highest SES, shown in green and Four = lowest SES, shown in red).

Several indictors have been chosen to reflect socioeconomic status (SES) in previous studies, including education, income, occupation, and race/ethnicity [7,14,21,22,23]. To further capture the multidimensional feature of SES, some studies used composite indices composed of multiple indicators to represent an individual’s socioeconomic advantage [24,25,26]. However, many of those indices were not officially adopted by local government, and aggregating SES may introduce additional bias when interpreting. In this study, we used a signal measure indicator, the median monthly household income (MHI) of a deceased person’s residential area, as a proxy to reflect the one’s socioeconomic status (SES). Income is the most widely used SES indicator, which directly and indirectly affects access to goods and services for health. In the 2011 Hong Kong census, the territory of Hong Kong was divided into 289 small areas, which are also known as Tertiary Planning Units (TPUs). To minimize issues regarding privacy, TPUs with a population less than 1000 were merged into the nearby TPUs to form 209 TPU clusters. One TPU was intentionally excluded due to the entire population within the area being prisoners, and therefore 208 TPU clusters were included in the final analysis. All TPU clusters were then grouped into four levels using the quartering method according to its MHI (52 TPU clusters in each group, where 1 indicates the group with highest SES and 4 is the lowest SES group), to evaluate whether there were differences in the effect of temperature on all non-accidental and cause-specific mortality (Figure 1). Each death record was linked with an SES group code according to his/her residential area by the TPU code.

### 2.4. Statistical Analysis

Distributed lag linear and non-linear models (DLNM) were performed to determine the association between daily mortality and ambient temperature for each SES group. As daily mortality counts generally follow an overdispersed Poisson distribution, a distributed lag model with a quasi-Poisson regression was used to evaluate the health effect of heat and cold while adjusting for the temperature at different lag days. The model used the following formula:

$$\begin{array}{cc} \mathrm{Log}\left[E\left(Y_{t}\right)\right] & =\alpha +\mathrm{cb}\left({\mathrm{Temp}}_{t}\right)\;+\mathrm{ns}\left(\mathrm{Humidity},\mathrm{df}=4\right)\\ & \;\;\;\;\;\;\;+\mathrm{ns}\left(\mathrm{Pressure},\;\mathrm{df}=4\right)+\mathrm{ns}\left(O_{3},\;\mathrm{df}=4\right)\\ & \;\;\;\;\;\;\;+\mathrm{ns}\left({\mathrm{SO}}_{2},\;\mathrm{df}=4\right)+\mathrm{ns}\left({\mathrm{NO}}_{2},\;\mathrm{df}=4\right)+\mathrm{ns}\left({\mathrm{PM}}_{2.5},\;\mathrm{df}=4\right)+\mathrm{ns}\left(\mathrm{Time},\;\mathrm{df}=9\times 7\right)+{\mathrm{Dow}}_{t}+{\mathrm{Holiday}}_{t} \end{array}$$

where α is the intercept; cb is the crossbasis matrix built up with *dlnm*() package in R; ns(): smoothing function using a natural cubic spline. A quadratic B-spline with three internal knots placed at the 10th, 75th and 90th percentiles of temperature was used to model the exposure–response relationship and a natural cubic spline was fitted with three internal knots placed equally spaced in the log scale to model the lag–response association [27]. 4 degrees of freedom (df) were used for potentially confounding metalogical and air pollutant variables. The model uses a maximum lag period of 21 days to capture the long delay of effects of cold and to exclude deaths that were advanced by only a few days (harvesting effect) [28,29]. The long-term and seasonality effect was controlled using 7 df per year. Day of the week (Dow) and local public holidays were also adjusted in the model. The Akaike Information Criterion for quasi-Poisson (Q-AIC) values were used for the choice of the degree of freedom.

The relationship between temperature and mortality was summarized as the exposure–response curve of relative risk (RR) accumulated across all lags. Minimum mortality temperature (MMT), with its 95% confidence interval (95% CI), was calculated. RR for total death and each SES group were estimated. Subgroup analyses for gender, age group (0–65 years and 65 years above) and cause-specific death (cardiovascular, respiratory and cancer) were performed. The results show that the MMTs for each subgroup analysis were close to 27 °C, so that was chosen as the fixed reference temperature to compare the temperature effect on each SES groups. Modelling choices were tested by a sensitivity analysis using different degrees of freedom for temperature and long-term trends, and length of maximum lag period. All analyses were performed using R project for statistical computing (version 3.6) packages *dlnm* and *mgcv* [30,31].

## 3. Results

### 3.1. Data Description

Hong Kong is a subtropical city with hot summer and mild winter. The mean and median daily mean temperature during the study period were 23.5 °C and 24.7 °C, with a range between 8.4 and 32.4 °C, respectively (Table 1). As a coastal city, the relative humidity is relatively high, with a median of 79%. Overall, 362,957 non-accidental deaths were included in the analysis after excluding cases without a valid TPU code (1.34%). There were more deaths in the lower SES group than higher SES in Hong Kong, with daily mortality ranging from 0 to 26 cases in the highest SES group and 14 to 192 cases in the lowest one (Table 2). Figure 1 shows the distribution of the TPU cluster by SES groups, of which the highest cluster (Deep Water bay, HK$178,000 or US$22,960/month) has reported 20 times higher median household income compared to the lowest (Tai O, HK$8000 or US$1031/month), indicating a large SES disparity among districts in Hong Kong.

### 3.2. Main Findings

**Figure 2:**
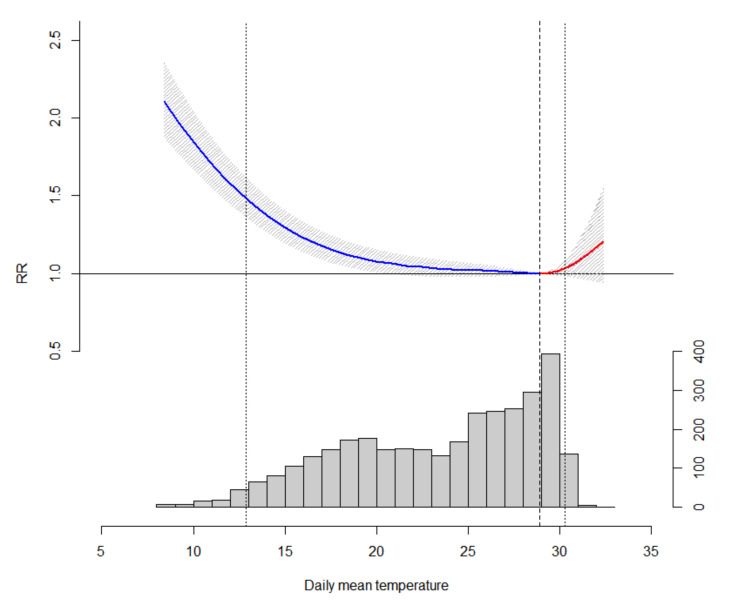
The cumulative effect (lag 0–21 days) of daily mean temperature on non-accidental mortality during 2007 and 2015 with the distribution of daily mean temperature at the bottom (number of days). The dashed in the middle indicates the minimum mortality temperature (28.9 °C) the two vertical lines on the sides indicates the 2.5th (12.9 °C) and 97.5th (30.3 °C) percentile of the daily temperature during study period.

**Figure 3:**
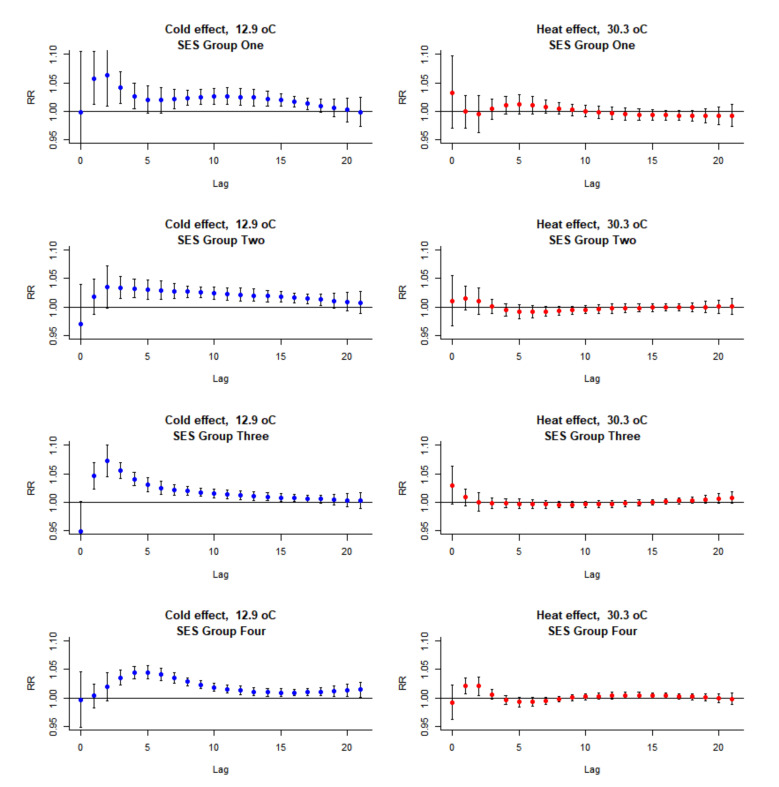
The Lag–response effect distribution during the 21 days lag period after exposed to a low (12.9 °C, 2.5th percentile) and high (30.3 °C, 97.5th percentile) temperature compared to reference temperature (27 °C).

As shown in Figure 2, the cumulative effect (lag 0–21) of cold posed a stronger effect on mortality when compared to heat with the reference temperature of 28.9 °C—the temperature with minimum mortality (MMT). When an extremely low temperature occurs (12.9 °C, 2.5th percentile of daily mean temperature), the mortality rate is almost 1.5 times higher compared to 27 °C. No significant overall heat was identified in the lag period of 0 to 21 days. For the lag–response effect, the hot effect tended to be more pronounced in the first 3 days with no harvesting effect. Meanwhile, the cold effect may last up to 3 weeks after the low temperature occurs (Figure 3). The cumulative cold effect in relative risk for lag 0–21 day was 1.463 (95% CI: 1.362, 1.571) and the heat effect for lag 0–3 days was 1.035 (95% CI: 1.011, 1.059) (Table 3). No overall clear harvesting effect in mortality was observed for both heat and cold effect.

**Figure 4:**
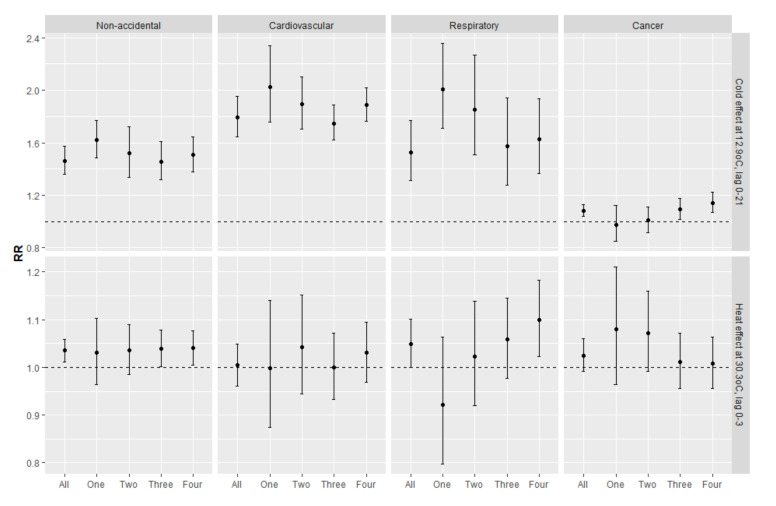
The cold (12.9 °C, 2.5th percentile at lag 0–21) and heat effect (30.3 °C, 97.5th percentile at lag 0–3) compared to 27 °C by cause of death and SES groups (where One = the highest SES, Four = lowest SES).

To compare the effect of high and low temperature among SES groups, the RR for each SES group were estimated (Figure 4). For non-accidental death, heat effect was found to be only significant in lower SES groups (Group Three: 1.039 (95% CI: 1.001, 1.078), Group Four: 1.040 (95% CI: 1.005, 1.076), while the cold effect was found to be significant across all SES groups (Figure 4).

For cause-specific temperature on mortality (Figure 4), the high temperature was found to be associated with significantly higher respiratory death, particularly among groups with the lower SES, whereas heat effect was not significant for cardiovascular deaths. On the other hand, the cold impact was significant for both cardiovascular and respiratory death and the effect was found to be generally higher in all SES groups. The results also suggested that the heat effect for respiratory death was higher than cardiovascular, whereas the cold effects were stronger for cardiovascular death than for respiratory death. The effect of cold temperature on cancer mortality was significant, although the effect size was much smaller than other causes. The cold effect on cancer deaths was found to be higher in lower SES groups, whereas the more affluent groups were not significantly affected. High temperature was found to be associated with increased mortality risk for cancer deaths.

**Figure 5:**
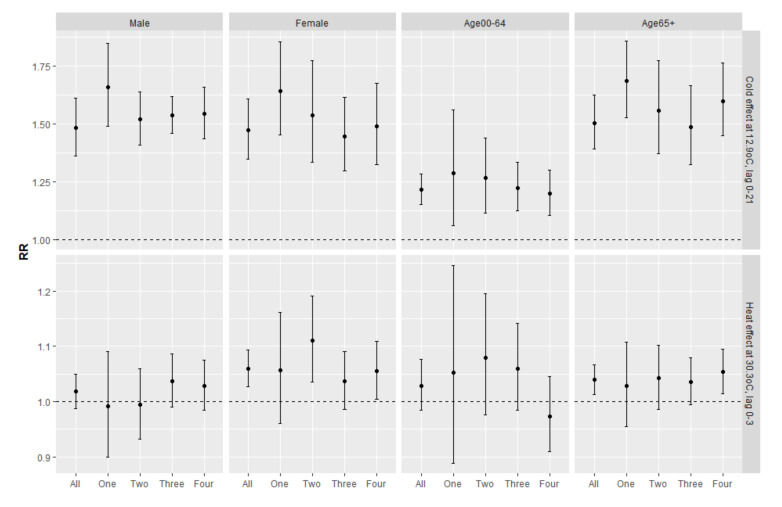
The cold (12.9 °C, 2.5th at lag 0–21) and heat effect (30.3 °C, 97.5th at lag 0–3) on non-accidental deaths compared to 27 °C by age, gender and SES groups where One = the highest SES, Four = the lowest SES.

Furthermore, the high temperature was associated with higher mortality risk among females than males, but no clear gender difference was observed for cold effect (Figure 5). Compared with the younger population, older individuals were generally associated with higher temperature-related mortality, especially for low temperature. Significantly higher cold-related mortality risk was observed in all SES groups, but heat-related mortality risk was only identified in the lowest SES group.

## 4. Discussion

This study is the first study to investigate the SES-related disparities on the mortality risk associated with the high and low temperature in Hong Kong. A J-shape temperature–mortality association was identified for this subtropical urban metropolitan city. Results are consistent with previous studies, suggesting that the cold effect is stronger than heat effect in most populations across many countries [27,32,33], and in subtropical cities such as São Paulo [34], Guangzhou [29] and Taipei [27]. The finding provides supporting evidence to the hypothesis that population living in a warmer climate are more adapted to cope with high temperatures, and more susceptible to cold weather.

The minimum mortality temperature in Hong Kong was found to be 28.9 °C, at 82% percentile of the daily mean temperature in the study period, which was generally consistent with the finding from other studies conducted on population with similar climate [21]. One study examined the relationship between temperature and mortality in 66 Chinese communities found that the MMT was 27.4 °C in southern China, which is the highest compared to other regions in China [35]. Country-wide and worldwide studies found that the MMTs are usually higher in warmer regions [27,33,35,36]. Moreover, the MMT identified in this study was slightly higher than the mortality threshold (28.2 °C) reported in a previous local study [21] which used the data from 1998–2006. One assumption could be that the population are adopting the increasing temperature with the effect of climate change [3,37,38], leading to a slightly higher tolerance of hot weather. With the effect of climate change, some studies anticipated that heat-related mortality will increase and eventually compensate for the reduction in cold-related death after 2050 [39]. Other studies suggested that despite the fact that the cold burden will be reduced by the relative effect, heat-related death will remain high across the entire 21st century [40], and the net effect may be inconsistent and subject to local context [41,42], indicating that it is still too early to neglect the health impact of cold weather in the coming decades.

The climate in subtropical regions typically has a very hot summer and less harsh winter. Residents are usually acclimated to a high temperature, which was considered as an important reason why an overall insignificant effect of heat was observed in this study [29,43]. In Hong Kong, the air condition was commonly installed in almost all indoor areas and public transports. The actual exposure of the population could be substantially reduced. However, the study found that the 3-day cumulative heat effect was significantly higher among areas with lower SES, which was also reported in some local and international studies [21,44,45]. A possible reason may be due to the characteristics of housing. The buildings in low-income areas tend to have a higher proportion of old buildings with poor ventilation and insulation. Hong Kong is a city with the highest housing price in the world, and individuals who suffer from poverty usually live in subdivided or temporary dwellings [46]. Those dwellings are often small with poor ventilation, in which heat can be easily trapped inside.

This study found that the cold effect was significant across all SES groups for non-accidental, cardiovascular, respiratory and cancer death. The result also shows a counterintuitive pattern that communities with higher SES were associated with higher cold-related mortality risk, which despite the differences was not statistically significant. Although this kind of analysis was rarely conducted in previous studies, some reported similar results [15]. In Hong Kong, communities with lower SES tends to have higher living density and stronger urban heat island effect [47]. This may suggest that the urban heat island effect may have potential benefit against cold weather in highly urbanized populations, which has been reported recently elsewhere [48]. The universal impact of low temperature on mortality may also be due to the low prevalence of central heating. Despite the high air condition coverage in Hong Kong, most air conditioning devices do not have heating functions and buildings were not designed to restore heat.

A stronger cold effect was identified on cardiovascular than respiratory mortality, which was consistent with previous local and international studies [34,49]. Previous physiological studies suggested that exposure to low temperature may cause elevated blood pressure [50,51], blood viscosity [52], plasma cholesterol and the tendency of blood clot formation in the vessels [53]. Changes in those risk factors may subsequently increase the risk of cardiovascular death. When a high-temperature event occurs, the risk of respiratory death increases as the SES decreases and only individuals living in the lowest SES group were significantly associated with higher risk. A similar pattern was also generally observed for cardiovascular death, despite the effect for all SES groups not being statistically significant. Some earlier local and international studies indicated that the area with lower SES was disproportionally affected by a higher concentration of air pollutants [54,55] and may pose both short and long term adverse effects on health [56,57]. A US study also suggested that in a large city, the exposure of NO_2_ concentration is significantly higher for individuals with lower household income [54]. A local study showed that areas with a high level of social deprivation were associated with higher exposure to PM_2.5_ [24]. Furthermore, the effect on air pollution on mortality may also be modified by that temperature. Some studies found that an adverse effect of particulate matter on mortality is stronger under hotter weather in Chinese and European populations [58,59]. The disparity further supports that individuals living in a lower SES community had a higher relative risk after exposure to hot weather, especially for respiratory deaths. This study also identified that cold temperature was associated with significantly higher cancer mortality risk. Several recent studies suggested that cold temperature could be an independent risk factor for cancer [60]. Cold exposure may increase metabolic stress, may contribute to tumorigenesis and higher cancer deaths [61]. A study used data from 166 countries and found a positive association between cancer mortality rate and serum average total cholesterol, which could act as a mediator of cancer development [62]. Unlike other causes, a significant cold effect was only observed in groups with lower SES. higher deprivation and lower-income have been linked with higher cancer incidence and mortality rate due to inequalities on lifestyle, environmental factors and access to services [63,64]. However, the cold effect on cancer and the role of SES have been underreported in current literature, which may be a potential gap for future research.

Significantly higher cold-related mortality risk was found for both males and males, and gender difference was found to be minimum for cold effect. However, females were found to be more susceptible to high temperature. Such a pattern has also been found in a local study [21] and studies elsewhere [8,65]. Older age is a well-known factor associated with higher susceptibility to temperature-related mortality risk [8,29,66,67], and this study found no exception, especially for low temperatures. No clear intra-SES group heterogeneity was identified in both age groups for low temperature effect. However, older persons in the lower SES group were significantly associated with higher heat-related mortality risk, whilst high temperature only posed a very minimum effect to their counterparts in better-off groups. A UK study found that lower SES was associated with a higher uptake of protective measures when experiencing hot weather [68]. A local study indicated that low-income individuals did not have the same level of protective measures, and that some may face financial constraints and still have to work outdoors under hot weather [69]. However, a recent local study found that higher income and education level were not associated with a higher prevalence of protective behavior during cold weather, and vulnerable groups such as the older population commonly underestimated their health risk [70], which partially explained the universal cold effect across all SES groups.

In the UK and Spain, cold weather has been recognized as a major public health concern at the national level, even though the weather during the winter in the two countries is generally milder compared to many other European countries. Nevertheless, the health impact of cold weather has not received adequate attention in Hong Kong, and only limited public services are made available when low temperatures occur [71]. Future policy should consider establishing a holistic strategy to enhance the protective measures at both individual and community levels and reduce the mortality and morbidity associated with low temperature.

A limitation of this study, which is shared by many other researchers of this kind, is the selection of SES measures. The small area level SES may not directly reflect the SES at the individual level. Future multilevel studies with both small areas and individual measures should be conducted, to further understand the effect of SES on the association between temperature and adverse health outcome. Moreover, the actual temperature exposure may vary from person to person, and outdoor ambient temperature may not reflect the exposure if a person spends more of the time in the indoor environment. Some behavioral and physiological factors such as lifestyle and pre-existing diseases may not necessarily be associated with SES, but have also proven to be related to personal exposure and the outcome of extreme temperatures. 

## 5. Conclusions

Despite the fact that Hong Kong has a milder winter climate, the overall cold effect was found to be much stronger and last for a longer period than the heat effect on mortality in Hong Kong. The subpopulation with lower SES is more vulnerable to high temperature, but low temperature universally affects the entire population across all SES groups in Hong Kong. A higher cold effect was observed in groups with the highest SES, despite the different was not statically significant. The heat island effect is a risk factor for heat-related deaths, but it may provide a protective effect during cold weather. This study raises the concern of the health effect of low temperature in subtropical regions, which should be recognized as an important public health issue at territory level. Strategies on mitigating the cold effect should not only focus on populations with lower SES; additional public services should be provided for different socioeconomic classes in Hong Kong.

## Figures and Tables

**Table 1:** Descriptive statistics of metalogical and air pollutant variables.

	Percentile							
Variables	Mean	Min	5th	25th	50th	75th	95th	Max
Mean Temperature (°C)	23.5	8.4	14.2	19.2	24.7	28.1	30.0	32.4
Mean Relative Humidity (%)	78.1	29.0	58.0	73.0	79.0	85.0	95.0	99.0
Atmospheric pressure (hPa)	1012.7	992.2	1002.6	1007.9	1012.7	1017.7	1023.1	1029.8
NO_2_ (μg/m^3^)	41.2	11.4	28.2	38.3	48.4	61	83.8	152.5
O_3_ (μg/m^3^)	51.2	4.9	13.6	21.7	36.9	56.1	85.4	139.3
SO_2_ (μg/m^3^)	13.9	3.2	5.6	8.4	11.8	16.9	28.9	80.6
PM_2.5_ (μg/m^3^)	31.4	4.9	9.2	15.8	27.5	42.5	67.5	138.5

**Table 2:** Descriptive statistics of daily mortality count in the study period.

SES Groups	Percentile							
	Min	5th	25th	50th	75th	95th	Max	Total
One—Highest SES	0	4	7	9	11	15	26	30,169
Two	3	10	14	18	21	26	38	63,808
Three	6	23	30	34	39	47	67	125,750
Four—Lowest SES	14	34	42	48	55	67	92	143,230
Total	31	86	99	109	120	143	192	362,957

**Table 3:** The relative risk (RR) for cold (12.9 °C, 2.5th percentile at lag 0–21) and heat effect (30.3 °C, 97.5th percentile at lag 0–3) compared to 27 °C with 95% confidence interval.

	Non-Accidental		Cardiovascular		Respiratory		Cancer	
SES Group	Cold	Heat	Cold	Heat	Cold	Heat	Cold	Heat
One	1.622 * (1.485, 1.771)	1.031 (0.964, 1.103)	2.026 * (1.755, 2.339)	0.998 (0.874, 1.140)	2.009 * (1.713, 2.356)	0.921 (0.797, 1.064)	0.976 (0.850, 1.121)	1.080 (0.964, 1.211)
Two	1.518 * (1.337, 1.723)	1.036 (0.985, 1.089)	1.893 * (1.707, 2.100)	1.043 (0.945, 1.152)	1.851 * (1.510, 2.268)	1.023 (0.920, 1.138)	1.009 (0.916, 1.111)	1.072 (0.991, 1.160)
Three	1.457 * (1.320, 1.607)	1.039 * (1.001, 1.078)	1.748 * (1.619, 1.888)	1.000 (0.933, 1.071)	1.575 * (1.278, 1.940)	1.058 (0.977, 1.145)	1.094 (1.018, 1.176)	1.012 (0.956, 1.071)
Four	1.506 * (1.378, 1.645)	1.040* (1.005, 1.076)	1.888 * (1.764, 2.019)	1.030 (0.969, 1.095)	1.626 * (1.364, 1.938)	1.099 * (1.022, 1.182)	1.142 * (1.068, 1.223)	1.008 (0.955, 1.064)
All	1.463 * (1.362, 1.571)	1.035 * (1.011, 1.059)	1.794 * (1.645, 1.955)	1.004 (0.961, 1.048)	1.525 * (1.312, 1.772)	1.049 * (0.999, 1.101)	1.081 * (1.037, 1.127)	1.025 (0.991, 1.060)

* Statistically significant at α ≤ 0.05.
